# Is relevant postpartum maternal psychopathology on the prognosis of psychomotor development in infants born after a threatened preterm labour across preschool ages?

**DOI:** 10.1192/j.eurpsy.2024.1693

**Published:** 2024-08-27

**Authors:** J. Andreu, J. Buesa, F. Ghosn, A. Moreno, L. Campos, B. Almansa, C. Zapata, M. Lizarán, N. Gómez, A. García-Blanco

**Affiliations:** ^1^Psychiatry, Hospital Universitario y Politécnico La Fe de Valencia; ^2^Psychology, La Fe Research Institute; ^3^Psychology, Hospital Universitario y Politécnico La Fe de Valencia, Valencia, Spain

## Abstract

**Introduction:**

Threatened preterm labour (TPL) is associated with long-lasting neurodevelopmental challenges, independent of prematurity. For instance, it is known that infants born a TPL show delayed communication and socio-individual skills, regardless of the gestational age at birth. Furthermore, TPL constitutes an adverse prenatal event that can induce maternal anxiety or depression, even during postpartum period, which can produce a deleterious effect of the prognosis of infant’s psychomotor development.

**Objectives:**

This study aimed to explore the influence of maternal psychopathology as well as other peripartum variables on the course of psychomotor development in children born after a TPL between the ages of 2 and 6.

**Methods:**

In this prospective cohort study, 117 mother–child pairs who experienced TPL were recruited. Psychomotor development was assessed using the Ages & Stages Questionnaires-Third edition at age 2 and 6. A regression model was carried out, including gestational age at birth, maternal anxiety trait, maternal history of psychological traumas, prenatal and postnatal maternal depression, anxiety, and cortisol as well as parenting stress as predictors.

**Results:**

Low gestational week at birth emerged as the most relevant factor in the course of increased communication delay (p < 0.001). However, parental psychopathology during prenatal or postnatal stages was not a relevant factor in the prognosis of Communication skills or Socio-Individual development.

**Conclusions:**

Gestation age at birth rather than parental psychopathology during peripartum period was the most relevant predictor of the course of psychomotor development between 2 to 6 years of age. Further studies should examine other potential modifiable predictors to moderate the impact of gestational age on psychomotor development.

**Disclosure of Interest:**

None Declared

